# Optical and motor changes associated with lighting and near vision tasks in electronic devices

**DOI:** 10.16910/jemr.16.2.3

**Published:** 2023-05-01

**Authors:** Elvira Orduna-Hospital, Ebrahim Safarian Baloujeh, Rafael Navarro, Ana Sanchez-Cano

**Affiliations:** Departamento de Física Aplicada, Universidad de Zaragoza; INMA, Consejo Superior de Investigaciones Científicas & Universidad de Zaragoza

**Keywords:** eye movement, eye tracking, reading, blinks, saccades, fixations, electronic reading devices, ambient lighting conditions

## Abstract

**Purpose:** To assess optical and motor changes associated with near vision reading under different controlled lighting conditions performed with two different types of electronic screens. **Methods:** Twenty-four healthy subjects with a mean age of 22.9±2.3 years (18- 33) participated in this study. An iPad and an e-ink reader were chosen to present calibrated text, and each task lasted 5 minutes evaluating both ambient illuminance level and luminance of the screens. **Results:** Eye-tracker data revealed a higher number of saccadic eye movements under minimum luminance than under maximum luminance. The results showed statistically significant differences between the iPad (p=0.016) and the e-ink reader (p=0.002). The length of saccades was also higher for the minimum luminance level for both devices: 6.2±2.8 mm and 8.2±4.2 mm (e-ink max vs min), 6.8±2.9 mm and 7.6±3.6 mm (iPad max vs min), and blinking rate increased significantly for lower lighting conditions. **Conclusions:** Performing reading tasks on electronic devices is highly influenced by both the configuration of the screens and the ambient lighting, meanwhile, low differences in visual quality that are transient in healthy young people, were found.

## Introduction

Computers and digital devices have an essential role in today’s world. First, these devices found their way into the working spaces, but now people spend hours staring at digital electronic screens outside the workplace too. These include, but are not limited to, mobile phones, tablets, e-book readers, gaming consoles, laptops, and other electronic devices. Since the development of the internet, individuals have had much easier access and dissemination of knowledge. Recent statistics indicate a figure of 66.2% as the penetration rate of the internet among people around the world (44). This has resulted in the digitization of paper books and the replacement of hardcopy printed documents with digital books. In this way, electronic books can be read on any device that has reading software.

Electronic ink (e-ink) and liquid crystal display (LCD) are the two main technologies used in display devices. LCD screens are multipurpose, have a higher refresh rate, and can display colors, which make them good choices for laptops and tablet PCs. On the other hand, the readability of screens on e-ink displays is improved, but colors cannot be displayed on e-ink readers (35). Despite this, compared to hardcopy materials, the contrast between the letters and background on these screens is less. Moreover, characters on a digital device are not as accurate or well defined, and due to the reflections and glare on these screens, viewing could be challenging (32). For viewing and reading on these devices, the eyes should be close to the screen and the crystalline lens should accommodate the formation of a clear image on the retina (13). Long-term use of these tools at close ranges might lead to the emergence of symptoms that include headache, blurred vision, eyestrain, dry eye, and diplopia that usually develop following near visual activities (3) and are closely related to ambient lighting and device setups (16; 46; 45; 48).

To evaluate visual discomfort, the visual process needs to be studied. Currently, eye-tracking is a technology developed to evaluate human interactions; screen-based eye-trackers are attached to the screen, the user sits in front of the screen, and they record ocular motility while a controlled task is performed. Nowadays, research with eye trackers is widespread due to the many objective parameters that can be measured. Specifically, recording reading tasks on electronic devices is gaining importance in objectively analyzing ocular motility (9; 12; 35).

Aberrometry is also an objective technique to measure the wavefront aberration changes of the eye, reporting the results as Zernike polynomials. There are different types of aberrometers, but the outgoing wavefront aberrometer based on Shack-Hartmann technology (21; 38) showed the best repeatability for total ocular aberrations, irrespective of microfluctuations in accommodation, instability of the tear film, and small eye movements (23; 34; 41). Blurred vision while viewing a computer is mostly correlated with accommodation (31); high-order optical aberrations (HOAs) have also been shown to significantly increase when blinking. This fact could be mostly related to both the quantity and quality of the tears (22).

There are few studies that have integrated measures of aberrometry before and after reading tasks with two different electronic reading devices in extreme conditions of maximum and minimum controlled lighting. In addition, in this study with these setting an attempt to objectify the visual discomfort has been made by measuring ocular motility with an eye-tracker, and the optical changes that occur in the visual system during reading with electronic devices. Other studies have been conducted to investigate the effect of viewing digital screens on the optical quality of the eye; the subjective method utilizes the asthenopia questionnaires for grading visual fatigue based on mental parameters (24) or another tool that is based on flicker changes, called visual fatigue meter (26). So, eye movement velocity, saccades, and eye blinks are other parameters that could be evaluated using eye trackers and could be considered indicators of the described visual discomfort, and they are the objective parameters used in this study.

Therefore, the aims of the present phenomenological research were first to develop an experimental design to measure ocular motility with an eye tracker during a short reading period of 5 minutes using an iPad and an e-ink reader and second to measure on-axis optical aberrations with a commercial Hartmann–Shack aberrometer to analyze visual quality before reading compared to after doing the task. The experiments were performed under two different ambient lighting conditions and two screen setups.

## Methods

### Participants

This prospective study included 24 healthy subjects with ages ranging from 18 to 33 years. It was approved by the Comité de Ética de la Investigación de la Comunidad de Aragón (CEICA) with reference PI21-074, and the conduct of the study adhered to the tenets of the Declaration of Helsinki. After an explanation of the nature and possible consequences of the study, written informed consent was obtained from all participants before the examination.

Full optometric evaluation was performed to the participants: measurement of the best corrected visual acuity (BCVA) both in distance and near vision, monocular accommodative amplitude, accommodative facility and vergence facility both monocular and binocular, dissociated and associated phoria and positive and negative fusional vergences both far and near, as well as fusion and stereopsis. Finally, ocular motility was also assessed. Patients who needed to wear their ophthalmic correction were asked to bring contact lenses because antireflective coating is done for the wavelength range from 400-700nm (Ibn-Elhaj & Schadt, 2001); reflectance for other wavelengths as that of the infrared used by the eye-tracker is higher (>750nm). The exclusion criteria to participate in the study were having any binocular problems, BCVA lower than 0.8 (20/25 on the Snellen chart) in one of the two eyes, suffering from some ophthalmic or systemic pathology that affected vision or having used electronic devices within one hour before the measurements.

### Materials

An e-ink reader (Ink pad 3, Pocketbook International SA, China) model PB740, with a screen size of 1404 x 1872 pixels, and an 8th generation iPad (Apple Inc., Cupertino, California, USA) Model A2270, with a screen size of 2160 x 1620 pixels, were used for the reading tasks. In both devices, a white background and black letter with a visual acuity of 0.8 were set and calibrated for the distance of 50 cm at which the reading task was performed (+2.00 D accommodative demand).

The eye-tracking device used in this study was the Tobii Pro Fusion eye-tracker (Tobii AB, Sweden), with a dual-camera system and two pupil tracking modes (bright and dark pupil), with dimensions of 374 x 18 x 13.7 mm and capturing gaze data at speeds up to 250 Hz. This eye-tracker Tobbi Pro Fusion maintains tracking robustness in different lighting environments and bright and dark pupil illuminators offer superior data regardless of eye shapes, ethnicity or age. To record the experiment, a camera equipped with a microphone (model AMDIS01B, Conceptronic, Germany) was also needed, which was directly connected to the laptop on which the Tobii Pro Fusion eye-tracker programs were installed: the eye-tracker Manager (Tobii AB, Sweden) for the device selection, and the Tobii Pro Lab (Tobii AB, Sweden) for calibration of the subjects in each reading were installed. The recordings and their subsequent segmentation were performed on this laptop.

The controlled maximum luminance, measured with a luminancimeter (Konica-Minolta, LS-160), of the e-ink reader (Emax) was 79.60 cd/m^2^ and that of the iPad (Imax) was 484.01 cd/m^2^, while the minimum luminance of the e-ink reader (Emin) was 0.14 cd/m^2^ and that of the iPad (Imin) was 1.56 cd/m^2^. Both reading devices were placed inside a controlled lighting cabinet to ensure optimal, repetitive, and correct lighting reaching the corneal plane of each participant in this study. To measure the conditions of maximum and minimum illumination applied in the reading plane and the corneal plane for each case, a calibrated (NIST traceability) spectroradiometer (model STN-BLK-C-SR, StellarNet, Inc. Tampa, Florida, USA) was used for analyzing the spectral power distribution in irradiance mode (µW/cm^2^) from 380 nm to 780 nm.

Inside the cabinet, a luminaire with white LEDs (6670K correlated color temperature) was used to achieve proper lighting levels, so 945.65 lx and 4.38 lx reached the reading surfaces at maximum and minimum lighting conditions, respectively; meanwhile, with these previous conditions, 216.82 lx and 1.32 lx were measured at the position where subjects would have their corneal plane during the reading tasks. In addition to the light provided by the cabinet according to the maximum lighting conditions and turning on the electronic devices, reaching the corneal plane, 264.15 lx was measured for the e-ink reader and 260.10 lx for the iPad. On the other hand, when the conditions of the cabinet were minimum illumination as well as minimum luminance of the devices, 1.63 lx for the e-ink reader and 1.62 lx for the iPad were obtained at the same corneal plane.

An IRX3 Shack-Hartmann device (Imagine Eyes, Orsay, France) was used to perform the aberrometry measurements under scotopic lighting conditions. This equipment has a near-infrared source (780 nm) to measure the shape of the wavefront, which is reflected out of the eye from a point source on the fovea. The outgoing wavefront is divided into several beams by an array of microlenslets, which produce spot images on a video sensor. The shape of the wavefront is determined by the displacement of each spot from the matching nonaberrated reference location (12; 21). After blinking, measurements were taken focusing on the Purkinje images obtained by aligning the instrument axis with the eye’s pupil (axial conjugation between an instrument 32x32 lenslet array and the eye’s pupillary plane). The manufacturer’s software calculates the aberrometry data automatically, fitting the measured wavefront of a selected pupil by the operator, 4 mm fixed pupil diameter, immediately after ending each five-minute reading task, as described previously. The real wavefront was analyzed with respect to the ideal wavefront to obtain the error of each measurement in terms of total root mean square (RMS Total), low-order RMS (RMS LOA), and high-order (RMS HOA).

### Procedure

The participants were asked not to use any type of electronic device at least one hour before the readings and not to perform close-up tasks so that it would not interfere with the baseline aberrometry measurements. Aberrometry was always performed by the same observer between 4:00 p.m. and 7:00 p.m. under scotopic conditions upon arrival of the participant before starting any reading, to serve as baseline measurements.

**Figure 1.**
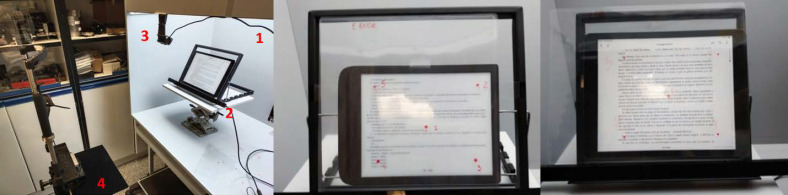
Left: Arrangement of the study elements. 1) Colour assessment cabinet. 2) Platform with the lectern where the eye-tracker and the reading device were placed. 3) Camera. 4) Chinrest. Center and right: Templates with the 5 calibration points placed on the e-ink reader (A) and on the iPad (B).

The participant stood with their chin and forehead resting on the chin rest 50 cm away from the reading device (e-ink reader or iPad) with the text calibrated for visual acuity of 0.8. The eye-tracker was placed just below the reading device 50 cm from the participant (Figure 1).

The subjects would take four readings of 5 minutes each according to the four randomized assumptions to avoid bias in the measurements due to adaptation to the light conditions: high ambient illuminance level (945.65 lx) with Imax luminance (484.01 cd/m^2^); high level of ambient illuminance (945.65 lx) with Emax luminance (79.60 cd/m^2^); low ambient illuminance level (4.38 lx) with Imin luminance (1.56 cd/m^2^); and low level of ambient illuminance (4.38 lx) with Emin luminance (0.14 cd/m^2^).

The electronic device to be used for the reading was selected from the eye-tracker Manager, and the calibrations were improved from the Tobii Pro Lab program; thanks to the camera, we could observe where on the screen the subject was looking at. For the calibration, we manually created a template for the iPad and another for the e-ink reader with 5 points on each. We place them on the reader, coinciding with the four corners and a central point of the screen, as shown in Figure 1. Therefore, during the calibration, the patient was asked to look at the points in the marked order (from 1 to 5). Once the eye-tracker was calibrated for the corresponding screen, the data collection by the eye-tracker started while the patient was reading aloud continuously. After 5 minutes of each reading task, the recording was stopped, and an aberrometry measurement was performed immediately afterward. There was a 15-minute break between readings in which the participant was prohibited from using electronic devices or performing close-up tasks.

### Data collection

**Figure 2:**
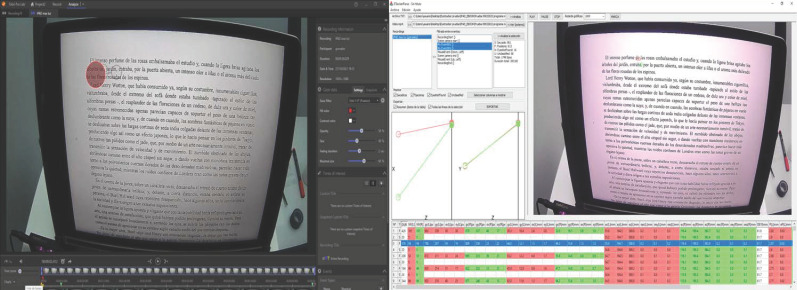
Left: “Event” segmentation of a recording of an iPad reading with the Tobii Pro Lab program. The lower green triangular marks are the start and end of the event. The red circle indicates where the subject is looking at that moment. Right: Analysis of the selected “event” of an iPad reading within the Etracker Parse program.

Each recording was reviewed and segmented with “events” in the Tobii Pro Lab program. To this end, it was marked when the subject began to read and again when exactly 4 and a half minutes had elapsed to close the “event”, which is what the program calls the selected time intervals between two marks (Figure 2). Once the events in the four recordings for each of the 24 participants were marked, the data from each recording individually (one for each reading) were exported to Excel (Microsoft® Office Excel 2016, Microsoft Corporation).

To manage the amount of data that Tobii Pro Lab exports, a specific custom-made program called Etracker Parse video (University of Zaragoza, Spain) was developed, with the help of which the parameters of interest were chosen: total reading duration (ms), number (n) of blinks, saccades and fixations, right eye (RE) and left eye (LE) pupil size (mm), length (mm) and velocity (m/s) of the RE and LE saccades separately, and mean saccadic and fixation duration (ms). These data were re-exported to Excel and grouped into a much more manageable database, with the variables of all of the recordings taken together for the statistical analysis.

### Statistical analysis

The measurements of the variables to be studied were recorded in an Excel database. Statistical analysis was performed using the Statistical Package for the Social Sciences (SPSS 25, SPSS Inc., IBM Corporation, Somers, NY, USA). The normal distribution of the values was examined with the Kolmogorov‒Smirnov test. Both aberrometric and eye-tracker parameters did not have a normal distribution, so the paired two-samples Wilcoxon test and Spearman’s correlation were used.

## Results

Twenty-four healthy subjects participated in the study, among which 16 were men (66.66%) and 8 were women (33.33%), with a mean age of 22.9±2.3 years (range 18-33) and a mean spherical equivalent refractive error of -0.75±1.50 D (range -4.50 to +2.50).

### Eye tracker measurements

Table 1 and Table 2 show the values of the different parameters measured by the eye tracker during the reading according to the same lighting conditions and different devices, as well as the comparison for the same device and different lighting conditions.

**Table 1.** Descriptive data and comparison of different environmental lighting conditions with the same device between the number of blinks, saccades and fixations, Right Eye (RE) and Left Eye (LE) pupillary diameters, length and velocity of the saccade in the RE and LE, and mean duration of saccades and fixations. Mean value±standard deviation (SD); minimum and maximum measured values, the differences are considered statistically significant (marked in bold) when a p-value <0.025 occurred.

	**iPad**			**e-ink reader**		
**Illuminance (lx)**	**Maximum** Mean±SD [min-max]	**Minimum** Mean±SD [min-max]	**p**	**Maximum** Mean±SD [min-max]	**Minimum** Mean±SD [min-max]	**p**
**Total duration (ms)**	270271.65±525.07 [269540-271416]	267170.80±13358.22 [210436-270668]	0.287	266334.33±13649.60 [210328-273220]	272335.50±8776.08 [269548-309558]	0.093
**Number of blinks (n)**	276.95±251.49 [37-881]	1189.20±2179.87 [15-7077]	0.445	251.09±202.51 [51-791]	496.00±818.05 [8-2704]	0.936
**Number of saccades (n)**	999.18±282.54 [454-1651]	841.30±799.61 [16-4131]	**0.016**	940.26±218.18 [659-1431]	610.85±320.72 [4-1243]	**0.002**
**Number of fixations (n)**	763.83±112.91 [400-959]	563.20±163.32 [11-732]	**<0.001**	747.63±95.19 [579-981]	483.75±228.30 [1-722]	**<0.001**
**RE pupil size (mm)**	2.74±0.35 [2.34-3.65]	5.39±0.68 [3.61-6.39]	**<0.001**	2.86±0.43 [1.97-3.65]	5.46±0.74 [3.53-6.39]	**<0.001**
**LE pupil size (mm)**	2.68±0.41 [1.98-3.56]	5.52±0.79 [3.42-6.67]	**<0.001**	2.79±0.44 [1.92-3.56]	5.43±0.78 [3.47-6.36]	**<0.001**
**RE saccadic length (mm)**	22.77±5.95 [15.02-41.18]	26.63±6.58 [14.08-39.16]	**0.020**	23.15±6.75 [14.48-43.62]	25.20±7.58 [11.15-38.67]	0.372
**LE saccadic length (mm)**	27.38±8.77 [13.42-49.00]	29.80±12.08 [9.26-53.01]	0.575	24.19±6.20 [11.30-39.24]	27.14±10.27 [12.03-52.83]	0.184
**Mean saccadic duration (ms)**	18.96±2.43 [13.99-22.91]	20.60±2.50 [16.44-24.65]	**0.010**	19.27±2.36 [13.16-23.02]	20.49±2.68 [15.80-25.27]	0.062
**RE saccadic speed (m/s)**	1.12±0.24 [0.81-1.75]	0.97±0.27 [0.43-1.52]	0.053	1.05±0.20 [0.70-1.68	1.09±0.49 [0.38-2.40]	0.276
**LE saccadic speed (m/s)**	1.11±0.24 [0.78-1.75]	1.13±0.53 [0.49-3.05]	0.444	1.18±0.29 [0.80-2.06]	1.01±0.26 [0.60-1.45]	**0.005**
**Mean fixation duration (ms)**	326.30±83.95 [235.46-653.85]	360.81±107.95 [116.36-587.60]	0.398	320.61±46.60 [218.70-403.30]	404.30±150.25 [120.00-682.60]	0.037

#### E-ink reader

According to the conditions under which the reading is carried out with the e-ink reader (Table 1), a greater number of blinks can be seen in Emin (496.00±818.05) than in Emax (251.09±202.51). Although the difference was not statistically significant (p=0.936), a large SD was observed in Emin, indicating the variability in blinking among the subjects.

Regarding the saccades, a greater number was reported in Emax (940.26±218.18) than in Emin (610.85±320.72), with statistically significant differences (p=0.002). Regarding the average saccadic length, for both eyes, it was greater in Emin (Table 1), but without significant differences from Emax, with a positive correlation but without statistical significance (Emin-Emax RE: r=0.463; p=0.053 and Emin-Emax LE: r=0.328; p=0.170). The saccadic speed remained practically constant, but the evaluation differentiating both eyes shows that while there are no significant differences (p=0.276) or correlation (r=0.415; p=0.069) in the RE, significant differences were found in the LE (p=0.005) with a positive significant correlation (r=0.545; p=0.019), being faster with Emax. The mean duration of the saccades was greater with Emin, bordering on statistical significance (p=0.062) and correlation (r=0.391; p=0.088).

There was a significantly greater number of fixations with Emax (747.63±95.19) than with Emin (483.75±228.30) (p≤0.001), but fewer fixations were observed with Emin because of their longer duration (Table 1) (p=0.037).

#### iPad

A greater number of blinks with Imin was observed (1189.20±2179.87) compared to Imax (276.95±251.49) but without significant differences (p=0.445) and with a high SD in the first case, with great variability in blinking between subjects when reading in low light.

The saccades occurred more under Imax (Table 1), and the differences were significant (p=0.016) compared to the e-ink reader. Likewise, their mean length for both eyes was greater in Imin, with significant differences for RE (p=0.020) and bordering significant positive correlation (r=0.451; p=0.053), while for LE, the p value was not significant (p=0.575) and without correlation (r=0.256; p=0.277), although it followed the same trend. The speed of these movements remained practically constant, with no differences between lighting conditions for both eyes (p>0.025) and without correlations (Imin-Imax RE: r=0.056; p=0.821 and Imin-Imax LE: r=0.442; p=0.058). The mean duration of the saccades was longer with Imin (20.60±2.50 ms) compared to Imax (18.96±2.43 ms), with statistically significant differences (p=0.010) and positive significant correlation (r=0.647; p=0.002).

On the other hand, there was a greater number of fixations with Imax (763.83±112.91) than with Imin (563.20±163.32); the difference was significant (p<0.001), but again, it was observed that the duration of these fixations was greater with Imin (Table 1) but this difference was not significant (p=0.398).

#### Emax vs. Imax

Comparing both devices for high lighting as shown in Table 2, a slightly higher number of blinks was observed when reading with the iPad (276.95±251.49) than with the e-ink reader (251.09±202.51), but without statistically significant differences (p=0.884).

A greater number of saccades was reported with the iPad (999.18±282.54) than with the e-ink reader (940.26±218.18), but this difference was not significant (p=0.506). Their mean length for both eyes remained practically constant with both devices, but when evaluating each eye separately, for the RE, no significant differences were observed (p=0.362) but a positive significant correlation was found (r=0.633; p=0.001), while for the LE, there were significant differences (p=0.014) and a positive significant correlation (r=0.766; p<0.001), being longer with Imax. Regarding the average speed for both eyes, it remained practically constant with both devices, but when evaluating each eye separately, there were no significant differences for the RE (p=0.287), but a positive significant correlation was found (r=0.523; p=0.010), while for the LE, there were considerable differences (p=0.024) and a positive significant correlation (r=0.800; p<0.001). The mean duration of the saccades was slightly longer for reading on the e-ink reader (19.27±2.36 s) than on the iPad (18.96±2.43 s), but there were no significant differences (p=0.761) and a positive significant correlation (r=0.635; p=0.001).

Evaluating the fixations, there was again a slightly higher number when participants read with the iPad (763.83±112.91) than with the e-ink reader (747.63±95.19), but without significant differences (p=0.212); the duration of these fixations was also slightly higher with the iPad than with the e-ink reader (Table 2), but the difference was not significant (p=0.372).

**Table 2.** Descriptive data and comparison between the two devices for the similar environmental lighting condition regarding the number of blinks, saccades and fixations, right eye’s (RE) and left eye’s (LE) pupillary diameters, length and velocity of the saccade in the RE and LE, and mean duration of saccades and fixations. Mean value±standard deviation (SD); minimum and maximum measured values, the differences are considered statistically significant (marked in bold) when a p-value <0.025.

	Maximum			Minimum		
Device	**iPad** Mean±SD [min-max]	**e-ink** **reader** Mean±SD [min-max]	**p**	**iPad** Mean±SD [min-max]	**e-ink** **reader** Mean±SD [min-max]	**p**
Total duration (ms)	270271.65±525.07 [269540-271416]	266334.33±13649.60 [210328-273220]	0.964	267170.80±13358.22 [210436-270668]	272335.50±8776.08 [269548-309558]	0.099
Number of blinks (n)	276.95±251.49 [37-881]	251.09±202.51 [51-791]	0.884	1189.20±2179.87 [15-7077]	496.00±818.05 [8-2704]	0.121
Number of saccades (n)	999.18±282.54 [454-1651]	940.26±218.18 [659-1431]	0.506	841.30±799.61 [16-4131]	610.85±320.72 [4-1243]	0.121
Number of fixations (n)	763.83±112.91 [400-959]	747.63±95.19 [579-981]	0.212	563.20±163.32 [11-732]	483.75±228.30 [1-722]	0.103
RE pupil size (mm)	2.74±0.35 [2.34-3.65]	2.86±0.43 [1.97-3.65]	**0.002**	5.39±0.68 [3.61-6.39]	5.46±0.74 [3.53-6.39]	0.687
LE pupil size (mm)	2.68±0.41 [1.98-3.56]	2.79±0.44 [1.92-3.56]	**0.002**	5.52±0.79 [3.42-6.67]	5.43±0.78 [3.47-6.36]	0.368
RE saccadic length (mm)	22.77±5.95 [15.02-41.18]	23.15±6.75 [14.48-43.62]	0.362	26.63±6.58 [14.08-39.16]	25.20±7.58 [11.15-38.67]	0.133
LE saccadic length (mm)	27.38±8.77 [13.42-49.00]	24.19±6.20 [11.30-39.24]	**0.014**	29.80±12.08 [9.26-53.01]	27.14±10.27 [12.03-52.83]	0.053
Mean saccadic duration (ms)	18.96±2.43 [13.99-22.91]	19.27±2.36 [13.16-23.02]	0.761	20.60±2.50 [16.44-24.65]	20.49±2.68 [15.80-25.27]	0.184
RE saccadic speed (m/s)	1.12±0.24 [0.81-1.75]	1.05±0.20 [0.70-1.68	0.287	0.97±0.27 [0.43-1.52]	1.09±0.49 [0.38-2.40]	0.913
LE saccadic speed (m/s)	1.11±0.24 [0.78-1.75]	1.18±0.29 [0.80-2.06]	**0.024**	1.13±0.53 [0.49-3.05]	1.00±0.26 [0.60-1.45]	0.513
Mean fixation duration (ms)	326.30±83.95 [235.46-653.85]	320.61±46.60 [218.70-403.30]	0.372	360.81±107.95 [116.36-587.60]	404.30±150.25 [120.00-682.60]	0.053

#### Emin vs. Imin

Comparing both devices for low lighting in Table 2, a higher number of blinks was observed when reading with the iPad (1189.20±2179.87) than with the e-ink reader (496.00±818.05) but without statistically significant differences (p=0.121), and with a large SD in both cases, yet there were alterations in blinking between subjects for cases when the reading task was performed under low light conditions.

Regarding the saccades, a greater number occurred with the iPad (841.30±799.61) than with the e-ink reader (610.85±320.72), without significant differences (p=0.121). The average length for both eyes was greater with the iPad than with the e-ink reader (Table 2), but there were no differences for any of the eyes (p>0.025) and high positive significant correlations (Imin-Emin RE: r=0.554; p=0.017 and Imin-Emin LE: r=0.903; p<0.001). The saccadic velocity remained practically constant with both devices, and the differences were not significant for any of the eyes (p>0.025), with high positive correlations in both eyes ((Imin-Emin RE: r=0.656; p=0.003 and Imin-Emin LE: r=0.758; p<0.001). The mean duration of the saccades was practically the same for both devices (20.60±2.50 s vs. 20.49±2.68 s for the iPad and e-ink reader, respectively, with p=0.184) with a positive correlation between them (r=0.865; p<0.001).

Concerning fixations, a higher number for reading on the iPad was found (563.20±163.32) than on the e-ink reader (483.75±228.30), but with no significant differences between devices (p=0.103); it was also seen that the duration of fixations for low lighting was longer for the e-ink reader (404.30±150.25 ms) than for the iPad (360.81±107.95 ms), without statistical significance (p=0.053).

#### Pupillary diameter

The expected results were obtained for the pupil diameter with both devices depending on the conditions under which the reading was performed (Table 1). The pupillary diameter of both eyes was considerably greater under minimum illumination than under maximum illumination. This is because the pupil is responsible for regulating the amount of light that reaches the retina. This difference was statistically significant when comparing lighting conditions for the e-ink reader (p<0.001) and for the iPad (p<0.001). The more lighting that reaches the retina, the more miotic the pupil becomes.

If we compare the devices (e-ink reader vs. iPad) under the same lighting conditions (Table 2), the pupillary diameter of both eyes is greater with Emax than with Imax. Although this difference is small, between 2.68 mm and 2.86 mm with a low SD, there were statistically significant differences between the reading devices (p=0.002). This could be related to the iPad's higher amount of luminance under maximum conditions. On the other hand, when comparing Emin vs. Imin, the pupillary diameter of both eyes was practically the same, with values between 5.39 mm and 5.52 mm for both devices, without significant differences among any of the eyes.

An attempt has also been made to model the pupil size according to one of the well-known formulas (43). Based on this paper and using our data the results in Table 3 were calculated. Computed results for iPad are coincident with those obtained performing the experiments. Meanwhile, theoretical results for e-ink could be questionable because they are too long, perhaps accommodation during the reading produces smaller pupils than expected. The results shown in Table 1 and Table 2 are smaller than the ones in Table 3 being more realistic than the calculated.

**Table 3.** Theoretical results of pupillary diameters calculated with the formulas of Watson & Yellott (43) for the different lighting conditions for iPad and e-ink.

23 years	Field (°)	L (cd/m^2^)	Pupil diameter formula (mm)
Imax	30	484.01	2.853
Emax	20	79.6	3.897
Imin	30	1.56	5.843
Emin	20	0.14	7.205

### Aberrometer measurements

**Figure 3.**
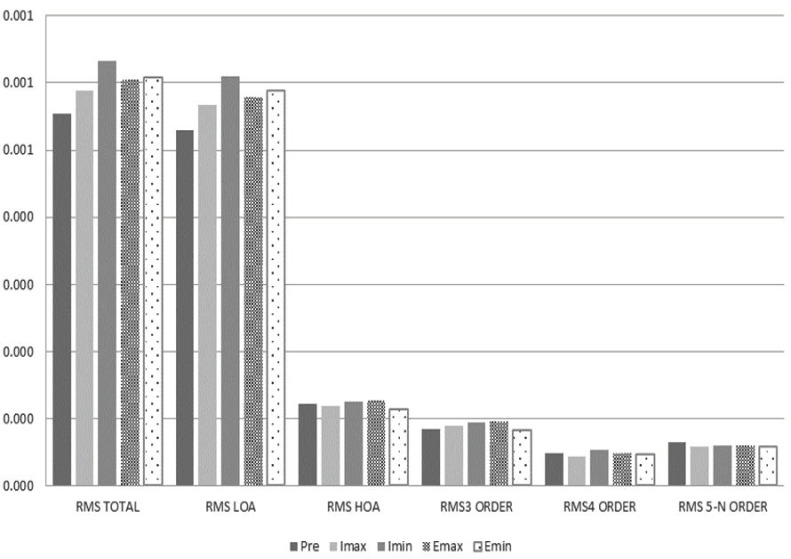
Comparison of the RMS (µm) measured before and after reading with e-ink reader and iPad in maximum and minimum lighting conditions. Baseline o previous (Pre) in black, iPad with maximum lighting conditions (Imax) in light grey, iPad with minimum lighting conditions (Imin) in dark grey, Ebook with maximum lighting conditions (Emax) in dark dotted, Ebook with minimum lighting conditions (Emin) in light dotted.

**Figure 4.**
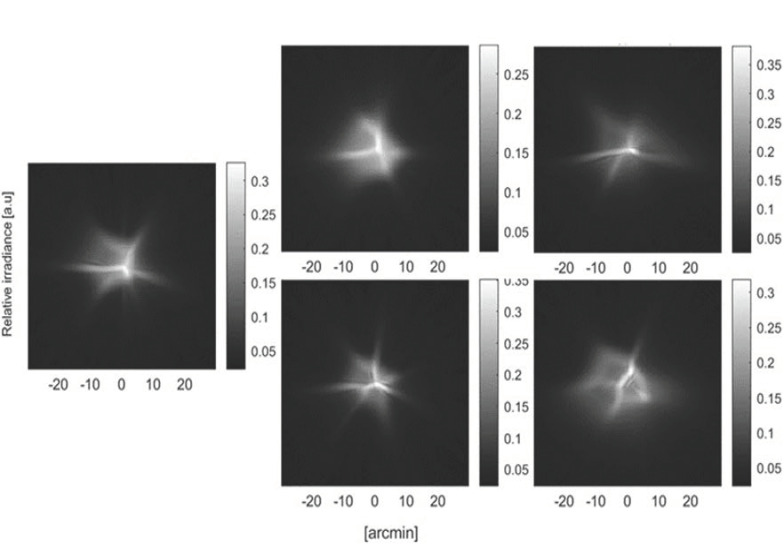
An example of Point Spread Function (PSF) obtained in the experiment. Left: Before reading task; center up: Emax; center down: Imax; right up: Emin; right down: Imin.

The aberrometic data followed non-normal distributions; therefore, nonparametric tests were used for statistical analysis between reference measurements and subsequent measurements after reading under different lighting conditions. Mean RMS ± standard deviation (±SD) for Total, LOA and HOA (3rd order, 4th order, and from 5th order) were evaluated. The analysis of the aberrometry results indicates that after 5 minutes of reading on both devices under all of the lighting conditions described before, with respect to the baseline measurements, there was an increase in ocular aberrations in general, although the RMS values had no statistically significant differences (p>0.05) (Figure 3), and individual variations could be found in the optical quality of the subjects (Figure 4). In the case of the Emax condition, greater differences were found in the RMS of the 3rd-order aberrations compared to the baseline, statistical significance (p=0.053).

**Figure 5.**
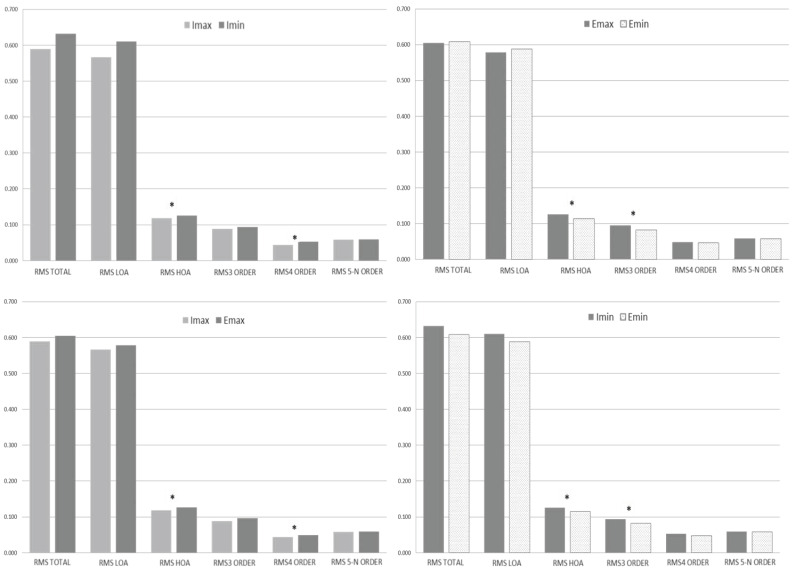
Comparison of the RMS (µm) of aberration measured after reading with e-ink reader and iPad in maximum and minimum lighting conditions. The asterisk indicates statistically significant differences (p<0.05). The iPad with maximum lighting conditions (Imax) in light grey, iPad with minimum lighting conditions (Imin) in dark grey, Ebook with maximum lighting conditions (Emax) in dark dotted, Ebook with minimum lighting conditions (Emin) in light dotted.

To complete the aberrometry analysis, the results after reading under the different lighting conditions on both electronic devices were compared. The analysis of the results in these post-reading situations reveals the differences as shown in Figure 5. On the iPad, aberrations, both RMS of LOA and HOA, increased under minimum lighting conditions; the RMS of HOA and specifically, those of the 4th order, were statistically significant. Changing the lighting conditions while reading on the iPad resulted in no-statistically significant differences for RMS when the lighting conditions varied from Imin 0.126±0.041 µm to Imax 0.118±0.039 µm (p=0.040). In this case, the statistically significant differences appeared in the 4th-order RMS values (p=0.027), and higher mean values were reported for Imin 0.053±0.027 µm compared to Imax 0.044±0.020 µm.

On the e-ink reader in Emin and Emax conditions, statistically significant differences could be observed in the RMS of HOA, specifically in the 3rd order with higher values in Emax than in Emin; meanwhile, RMS of LOA increased in the Emin condition although not in a statistically significant way. The same analysis was carried out on the e-ink reader after reading, and the RMS values of HOA were compared between the Emin (0.114±0.037 µm) and Emax (0.127±0.041 µm) conditions, bordering statistically significant differences (p=0.051). Analyzing various orders of aberration in more detail, the differences were found in the 3rd order, with values for Emin of 0.082±0.038 µm and Emax of 0.096±0.043 µm being higher in the latter case (p= 0.067).

The RMS of aberrations for the iPad and e-ink reader are shown and compared with maximum illumination. Setting the Emax induces greater ocular aberration than the Imax, and these differences were significant for the HOA, specifically in the 4th order. Under conditions of maximum illumination, the comparison of both devices indicates that there are differences between the RMS of HOA of the Imax (0.118±0.039 µm) and the Emax (0.127±0.041 µm); p=0.038. The HOA analysis indicates that these statistically significant differences appear in the 4th order, Imax (0.044±0.020 µm), with those of the Emax (0.049±0.021 µm), obtaining a value of p=0.031 and being higher for the e-ink reader.

The same analysis with the iPad and e-ink reader at minimum lighting is shown; in these reading conditions, greater aberrations appear for the Imin than the Emin, and statistically significant differences were found for the HOA, specifically in the third order. Under minimum illumination conditions, the HOA aberrations of Imin (0.126±0.041 µm) were compared with those of Emin (0.114±0.037 µm), obtaining a value of p=0.005. The 3rd-order aberrations of Imin (0.093±0.037 µm) were compared with those of Emin (0.082±0.038 µm), obtaining a value of p=0.018.

## Discussion

The present research achieved two main goals: to develop an experimental design for measuring ocular motility with an eye-tracker for the 5-minute reading task using an iPad and an e-ink reader and to measure on-axis optical aberrations for analyzing the visual quality before and after performing the task. Furthermore, the experiments were accomplished under maximum and minimum ambient and device lighting conditions.

Reviewing the literature, some studies associated lighting conditions and eye movements with visual fatigue. Benedetto et al. (2) found that when the ambient illuminance and the screen luminance were low, the number of blinks increased, and more saccades occurred, although slower, and the fixations were more frequent and of greater duration, and their pupillary diameters were larger. In addition, the tear evaporates less rapidly when blinking more frequently, so it helps to reduce visual fatigue. This is similar in almost all aspects to our study, confirming that in low ambient lighting and low luminance, the number of blinks and fixations is higher, their duration is longer, and the pupillary diameter enlarges. In contrast, the number of saccades in our case was lower under low lighting, and the speed of these movements remained constant under low and high lighting conditions.

Regarding lighting conditions, it has been described that pupillary movements result from the equilibrium between the activity of the iris sphincter muscle, pupillary contraction, which is innervated by the parasympathetic nervous system, and the movement of the iris dilator muscle innervated by the sympathetic nervous system. The rod and cone photoreceptors send stimuli to intrinsically photosensitive melanopsin-containing retinal ganglion cells (ipRGCs) for the pupillary light response. Both types of photoreceptors, rods, and cones, are responsible for the initial constriction related to the pupillary light response (0.2–1.5 s); ipRGC cells are responsible for maintaining pupillary constriction and responding after stimulation. The spectral absorption and specificity of each of the four types of receptors imply that their responses and behavior are directly associated with the wavelength of light, intensity, angle, and duration of stimulation (11). Changing the lighting of the environment and the lighting profile of where the images will be shown can significantly alter pupillary measurements. Blue light causes greater pupillary reactions, and lower light intensity induces pupil dilatation. Lei et al. (19) stated that the pupillary response after stimulation increases monotonically with increasing stimulus intensity, ranging from 0.1 to 400 cd/m^2^. In this study, we observed a smaller pupil diameter with the iPad in maximum lighting conditions; this is consistent with findings in the bibliography, since the spectral profile of the iPad contains a greater amount of blue light compared to the e-ink reader, which can generate greater pupillary contraction.

Dynamic pupillometry in our study was performed using the eye-tracker by measuring the pupil diameter continuously during the five-minute visual task; it was performed under the previously described constant lighting conditions, and SD implied more fluctuations under low lighting conditions for both devices. It has been reported that the stimulus required to measure the response after stimulation of the ipRGC characteristics does not need to exceed 400 ms. This refers to the time it takes for rod and cone cells to achieve ambient lighting adaptation before pupillary measurement or adaptation between stimuli. Park et al. (27) and Bin Wang et al. (42) suggest that 10 and 20 minutes, respectively, of initial dark adaptation should be given before performing pupillary reflex tests. Ken Asakawa et al. (1) state that natural lighting is sufficient to capture the cone response with 5 min of light adaptation, and the rod response can be obtained after at least 10 min of dark adaptation.

The relationship between the frequency of blinks and visual fatigue has been studied in some research (4; 8). Their findings support that low blink frequencies cause greater ocular dryness and consequently greater fatigue over time, as stated by Benedetto (2). According to Li et al. (20), the lower the number of blinks during a task, the greater the fatigue of the subject, and the higher the number of saccades per second, the lower the discomfort. This is not in exact agreement with our study, since with lower ambient lighting and lower screen luminance of both devices, a higher number of blinks and a lower number of saccades occurred, although it is true that our low lighting conditions were quite extreme. It should be noted that during the study, the eye tracker encountered difficulties in the detection of the eye when setting the minimum luminance on both devices.

A reduced blink rate has been reliably documented during computer tasks, which ranges from a blinking rate of 18.4 blinks/min before computer use and during tasks of 3.6 blinks/min (28) or from 22 blinks/min while relaxed, reduced to 10 blinks/min while reading a book and dropping to only 7 blinks/min while reading text on a computer screen (39). The following have been reported during computer tasks or under varying test conditions: poor image, reduced contrast or font size, possible glare, or required cognitive jobs. Even with hand-held electronic devices located at closer distances and below eye level, lower blink rates were reported, which might be related to the gaze angle, but the cause is still unknown (37). Regarding the blinking rate, our results did not present statistically significant differences, as Talens-Estarelles et al. (36) described in their study. They showed that the blinking rate remained constant among four different displays: computer, tablet, e-reader, and smartphone, suggesting that the blinking rate could be related to cognitive demands rather than the display method, as other authors mentioned (6; 15; 33). Ambient lighting conditions with imbalanced luminance between the screen and its background or reflections from the digital device can cause discomfort and disability glare from the screen, reducing contrast and leading to inferior image quality (37). This degraded visual image of electronic screens has been associated with a reduced blink rate (7; 29). These results match our results, and based on them, we can say that reduced data were found depending on the lighting and the lower the lighting was, the higher the number of blinks.

Therefore, lighting conditions seem to be crucial in experiments where accommodative demands are needed. Van Ginkel et al (40) found refractive states, in terms of mean spherical equivalent (M), of -1.64 D for white light and -1.91 D for red light, both for an accommodative demand of 2.50 D, which is similar to the setting in this study. This myopic difference by wavelength reveals that choosing the target illumination is fundamental not only to the spectral power distribution but also to the intensity. The aberrometry and ocular motility results in this study also confirm this. Gomes JMR et al. (10) compared ocular aberrations after reading a printed sheet of paper versus reading on a computer screen under photopic conditions. In the case of reading with the computer, no significant differences were observed after comparing the previous and subsequent measurements of aberrations that were lower than the 5th order. On the other hand, on the printed sheet, they noticed significant changes in the 3rd order. However, when comparing the aberrations between reading the printed sheet and reading on the computer, they did not observe significant differences in aberrations lower than the 5th order. In the present study, no significant differences were observed between the measurement before and after each reading, while significant differences were found when participants read on the e-ink reader, which is similar to a printed page, and when they read on the iPad, since the aberrations were greater with the e-ink reader, being significant in the 4th order. Furthermore, ocular aberration can vary with accommodation, changes in illumination, and other psychophysical factors. Even under steady observing conditions, the optics of the human eye are not constant, exhibiting temporal instability in the form of fluctuations (30). The magnitude of these fluctuations could be up to 0.5D (5). Some studies have shown that fluctuations exist not only in the defocus error but also in other HOAs (14; 18; 30). These microfluctuations, both accommodative and pupillary, could be an important objective indicator of visual fatigue in sustained near work (47).

Regarding all of the aspects studied in this project, it can be pointed out that a greater number of saccades occurred, shorter in length and duration, both with iPad and e-ink reader in high lighting conditions, as well as a greater number of fixations and with shorter duration, indicating higher reading speed. When comparing both devices for high illumination, a greater number of fixations, saccades, and, above all, blinks were seen with the iPad; as well as the decrease in the size of the pupil due to the greater amount of light that reaches the retina. Although the HOAs are lower under these conditions and for this device, it may be due to the smaller pupillary diameter. Najmee et al. (25) found a relatively similar accommodation microfluctuation value when performing digital reading for 5 minutes with and without activating the night shift mode, associating this insignificant comparison between modes with a short duration of exposure.

In conclusion, our measurements indicate that after five minutes of the reading task, the HOA varied from the original state, increasing slightly after the tasks for all lighting conditions and types of digital format. These increases were not statistically significant, suggesting a quick recovery of the visual system in our study group, which consisted of young people, but it has not been particularly demonstrated, that the increase was related to reading or even to a near task. The setup with respect aberration measurement was insufficient to get meaningful results and the approach of measuring aberrations in this setup should be improved. Lighting conditions are crucial in these types of experiments; setting minimum luminance levels on the screens, the total number of saccades and their length, together with the number of blinks, were higher, implying more visual discomfort. Nevertheless, further experiments are needed using both eye trackers and aberrometers and more subjects of different age ranges to acquire robust, statistically significant trends related to lighting conditions and type of task.

### Ethics and Conflict of Interest

The authors declare that the contents of the article are in agreement with the ethics described in http://biblio.unibe.ch/portale/elibrary/BOP/jemr/ethics.html and that there is no conflict of interest regarding the publication of this paper.

### Acknowledgements

This research was funded by the Agencia Estatal de Investigación, Ministerio de Ciencia e Innovación of the Spanish Government (Grant PID2019-107058RB-I00 funded by MCIN/AEI/10.13039/501100011033), the European Union’s Horizon 2020 research and innovation programme under the Marie Skłodowska-Curie grant agreement No 956720, Gobierno de Aragón-Departamento de Ciencia, Universidad y Sociedad del Conocimiento-No LMP39_21, Fundación Ibercaja and University of Zaragoza No JIUZ-2021-CIE-03 and Ayudas para la adquisición de infraestructura de investigación (Modalidad A). Contrato Programa Plan De Inversiones e Investigación Gobierno de Aragón-Universidad de Zaragoza. Fondos Feder-2020.
